# Measuring the operational efficiency of individual theme park attractions

**DOI:** 10.1186/s40064-016-2530-9

**Published:** 2016-06-22

**Authors:** Changhee Kim, Soowook Kim

**Affiliations:** College of Business Administration, Seoul National University, Room# 515, SK Business Center(Bldg.58), Gwanakro 1, Gwanakgu, Seoul, Republic of Korea; College of Business Administration, Seoul National University, Room# 501, SK Business Center(Bldg.58), Gwanakro 1, Gwanakgu, Seoul, Republic of Korea

**Keywords:** Theme park, Attraction management, Management effectiveness evaluation

## Abstract

This study assesses the operation efficiency of theme park attractions using the data envelopment analysis, utilizing actual data on 15 attractions at Samsung Everland located in Yongin-si, Republic of Korea. In particular, this study identifies crowding and waiting time as one of the main causes of visitor’s satisfaction, and analyzes the efficiency of individual attractions in terms of waiting time. The installation area, installation cost, and annual repair cost are set as input factors and the number of annual users and customer satisfaction as output factors. The results show that the roller coaster-type attractions were less efficient than other types of attractions while rotating-type attractions were relatively more efficient. However, an importance performance analysis on individual attraction’s efficiency and satisfaction showed that operational efficiency should not be the sole consideration in attraction installation. In addition, the projection points for input factors for efficient use of attractions and the appropriate reference set for benchmarking are provided as guideline for attraction efficiency management.

## Introduction

A theme park is, literally, a “park with a theme,” and can be defined as a full-day tour site for families (Kyriazi 1976). Theme parks often have what is called “attractions,” which refer to rides that provide fun, out-of-the-ordinary experiences for visitors to enjoy, with the help of other thematic elements and equipment within the park. The goal of a theme park is to attract as many visitors as possible, however, it has been reported that crowdedness can deter more people from visiting the park. Neuts and Nijkamp (2012) found a negative relationship between perception of crowdedness to visitor number based on case-study of a city in Belgium, and this negative relationship was further studied by Eroglu et al. (2005), Miller and McCool (2003), and Mehta (2013), who found that consumers either give up using or tend to avoid facilities they know will be crowded. Furthermore, it has been noted that crowdedness does not only deter visitors, but also lower the satisfaction of those visiting: Manning and Ciali (1980)’s study on leisure activities showed that higher concentrations of people result in lower satisfaction, while Bielen and Demoulin (2007) confirmed that customer waiting is recognized as a negative experience by customers and lowers satisfaction.

Due to this dilemma, the significance of crowd control at theme parks has been continuously discussed, especially with regard to major attractions that expose customers to long waiting and subsequently, to a greater awareness of the crowdedness. Under the recognition that high crowdedness is a negative factor on customers’ satisfaction, this study investigates the case of Samsung Everland in the Republic of Korea to analyze the operational efficiency of attractions at theme parks. The efficiency of major attractions at Samsung Everland is analyzed to improve the performance of the theme park through data envelopment analysis (DEA), using the actual data on individual attractions for the input and output factors. The results of this study enabled the identification of efficient attractions from inefficient ones and also the cause of attraction inefficiency, which shed light on possible considerations for future theme parks to achieve maximum efficiency in their attraction configuration and also in the efficiency management of attractions. The implications of the analysis results are further discussed to take into account the discrepancy between operation efficiency and customer satisfaction in theme park attractions and to give suggestions on attraction configuration in future theme parks.

## Literature review and background

### Theme park’s physical environment

Hygiene factors have the role of providing information on service quality or product configuration to customers while improving their trust on the corresponding service at the same time (Wilson et al. 2012). As theme parks are defined, in addition to the definition provided by Kyriazi (1976) above, as attractions emphasizing a specific theme by creating a new atmosphere (Milman 1988), the visitor-attraction industry encompassing cultural and non-profit facilities altogether (Cameron et al. 1996), and facilities that adds a certain theme to existing enjoyment and recreational facilities (Milman 1988), it can be said that the hygiene factors at theme parks are its physical environment, which is where the providers’ service is delivered to customers and their interaction takes place (Baker et al. 1994).

Physical environment consists of many components, which are categorized using various methods in previous literature. Wakefield and Blodgett (1996) classified physical environment according to consumption purpose and facility use duration, while Robson (1999) grouped the physical environment at restaurants into ambient, design, and social factors in analyzing how the various components, such as music, lighting, table arrangement, furniture, materials, and so on, affected customer satisfaction. Related to theme parks, Dong and Siu (2013)’s study on two theme parks in Hong Kong used the term “servicescape,” which is an idea suggested by Bitner (1992) to express the environmental aspect in the field of service provision, and analyzed which essential aspects of servicescape affected visitors’ evaluation of the theme parks.

It has been reported that simulation inside physical environment affected both consumer cognitive and emotional reactions (Robert and John 1982). Specifically to theme parks, Kawamura et al. (2004) emphasized the role of attractions within the theme park’s physical environment, as attractions are the main factor determining visitor’s individual preferences of theme parks. Milman (2001) also highlights the importance of managing visitors’ perspectives through attraction management to meet their expectations of interactive adventure, fantasy, and mystery at theme parks. In this study, customer satisfaction survey results for individual attractions at Samsung Everland will be used in conjunction with the operational efficiency of the attractions to analyze how each attraction contribute to customer satisfaction in relation to its efficiency.

### Theme park’s operational efficiency

Customer satisfaction comes from the pleasant fulfillment of consumption experience and a evaluation process on the degree of consistency between pre-experience expectation and post-experience performance (Norvell 2012; Oliver 2014), which may affect the customer’s intention to revisit. Customers’ perception of risk can be a factor in this evaluation process, and in this sense, if uncertain or unsettling factors for customers are identified and well controlled, greater customer satisfaction be delivered to positively affect revisit intention. (Day 2003).

Because they are recreational facilities that depend on an extremely volatile visitor attendance, delivering high customer satisfaction is vital for theme parks and consequently, risk—in particular, functional risk—control also becomes crucial. In the case of theme parks, one of the functional risks that is most exposed to the customers’ perception is waiting time. Waiting time works as an important factor in the customer’s decision making process, along with cost, especially in service facilities such as theme parks (Greenleaf and Lehmann 1995), and previous studies show that waiting time is one of the factors that promotes a negative relationship (Bielen and Demoulin 2007) and conflict (Houston et al. 1998) between the service provider and the customers. However, the volatile visitor attendance makes it difficult for theme parks to properly predict the demand for individual attractions, which often lead to longer waiting time in theme parks (Luo et al. 2004).

There have been studies on efficiency of theme parks, such as Liu (2008) which conducted profitability measurement on theme parks in the United Kingdom, however, few to none can be found when the topic is narrowed down to the efficiency of theme park attractions. As this study was unable to benefit from previous studies in deciding the input and output variables, their selection referred to logical reasoning based on approaches used for traditional DEA models. In terms of input factors, Li et al. (2009) mentioned that traditional DEA approach sets the DMUs’ fixed costs as input factors, and Sueyoshi and Sekitani (2005) included variable inputs and fixed inputs as the two types of input variables in their DEA model. Based on such traditional approaches, this study uses installation area and installation cost (fixed costs) and annual repair cost (variable cost) as the input variable. In terms of output variables, Azadeh et al. (2008)’s use of quantitative and qualitative outputs was reflected in this study to use the number of annual users and customer satisfaction. The input and output variables used in this study are illustrated in Table 2.

### Theme park in Korea: Samsung Everland

Samsung Everland was established in 1976 in Yongin-si, South Korea by Samsung Corporation and is currently the largest theme park in the country, offering various attractions, seasonal parades, and festivals which are continuously updated with new contents based on the theme park’s long-accumulated knowhow. Not only is Samsung Everland the top theme park in Korea but it has also gained global recognition, with its being selected as the worlds’ top 4 theme park by Forbes in 2004 and ranked 8th among the top 100 Korean brand names by Brandstock.

A long list of accolades show the high customer satisfaction achieved by Samsung Everland, including number on ranking in the Korea Management Innovation Contest for Customer Satisfaction for 5 years in a row (1996–2000) as well as being number one in the Korea Service Quality Indication’s theme park segment for 14 years (2013), the Korean Net Promotoer Score’s Most Recommended Enterprise Award for the theme park segment for 7 years (2013), and the Korean Customer Satisfaction Index (KCSI) for the leisure segment for 20 years (2014). It also won the Presidents’ Prize at Korea Brand Awards (2003) and the Big E Awards for Parade (2005).

However, while customer satisfaction of the theme park is high, there still exist areas for improvement, especially in terms of attraction waiting time which scored the lowest for customer satisfaction among the various service elements at Everland in the Korea Consumer Agency (KCA)’s survey conducted on 1000 randomly-selected consumers at the theme park in 2012. In this context, this study investigates 15 attractions—*T Express*, *Double Rock Spin*, *Amazon Express*, *Let’s Twist*, *Rolling X*-*Train*, *Championship Rodeo*, *Flying Rescue*, *Lily Dance*, *Global Village*, *Sky Dancing*, *Flying Elephant*, *Peter Pan*, *Top Jet*, *Flash Bang Bang*, and *Royal Jubilee Carousel*—operated as of 2013 at Samsung Everland, as a case study in analyzing the efficiency of individual attractions at theme parks.

## Model

**Fig. 1:**
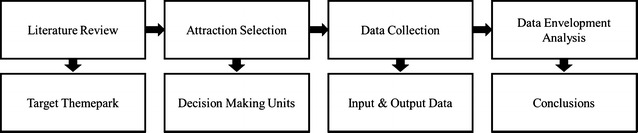
Research flow

The structure of the study is shown in Fig. 1. The target theme park is designated, Samsung Everland in this study, then 15 attractions at Everland were selected as the decision making units (DMUs) for operational efficiency assessment and their data were collected. The operational efficiency of the attractions were analyzed using data envelopment analysis (DEA).

### Linear programming and data envelopment analysis

**Fig. 2:**
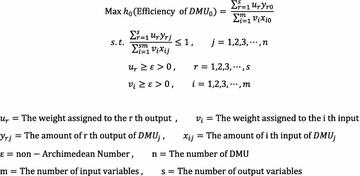
Fractional programming problem

Linear programming (LP) is a mathematical technique to optimize limited resource allocation for the achievement of decision making goals. The technique is mainly used for profit maximization or cost minimization issues and gives both the objective function and the constraints condition in linear forms (Papadimitriou and Steiglitz 1982). Data envelopment analysis (DEA) is based on such linear programming and was introduced by Charnes et al. (1978), who defines DEA as a linear programming technique to maximize the ratio of output weighted sum to input weighted total under the constraints condition that the ratio should not exceed 1 while each input factor and output factor’s weighted values exceeds 0. DEA is used to assess inefficiency levels of input/output factors based on ranking analysis, which is generally done by using the Charnes-Cooper-Rhodes (CCR) model or Banker-Charnes-Cooper (BCC) model for DEA where the difference between CCR and BCC models is that the BCC model accounts for returns to scale. As the purpose of this study is to utilize DEA to rank the relative efficiencies of the DMUs and to produce implications for enhancing the efficiency through the identification of the inefficiencies in input and output factors and the benchmarking target, the present study will utilize the input-oriented CCR Model assuming constant return to scale (CRS), which can be calculated as in Fig. 2.

### Input, output factors and decision making units

The 15 attractions at Samsung Everland selected as DMUs are listed in Table 1 including their installation cost and classification, and further information on the DMUs with photographs are given in the Appendix.

**Table 1:** Decision making units *Source*: http://www.everland.com

DMU	Attraction classification
T Express	Roller coaster type
Double Rock Spin	Thrill ride type
Amazon express	Water roller coaster type
Let’s Twist	Thrill ride type
Rolling X-Train	Roller coaster type
Championship Rodeo	Thrill ride type
Flying Rescue	Fall type
Lily Dance	Rotating type
Global Village	Water roller coaster type
Sky Dancing	Rotating type
Flying Elephant	Rotating type
Peter Pan	Rotating type
Top Jet	Rotating type
Flash Bang Bang	Fall type
Royal Jubilee Carousel	Rotating type

To find the relative efficiencies of the DMUs, DEA was conducted using the installation area, the installation cost, and annual repair cost of the attraction as input factors and the number of annual users and customer satisfaction as output factors. Of the input factors, the installation area and installation cost are fixed costs while the annual repair cost is a variable cost. The number of annual users, which is one of the output factors, represents how frequently the attraction was used each year and thus includes considerations of the waiting time. The other output factor, customer satisfaction, is based on the average of the attraction satisfaction results of the 2012 survey conducted by Samsung Everland’s Resort Business Headquarters.

With regard to the number of input and output factors and the number of DMUs, Nyhan and Martin (1999) postulate that the optimal number of input factors and output factors depends on the number of DMUs because in DEA, a greater number of input and output factors will result in a greater number of efficient DMUs, making it hard to identify inefficient DMUs. Thus, Banker et al. (1984) and Nunamaker (1985) both state that the number of DMUs should be at least three times more than the sum of the number of input factors and output factors. Further guidelines were given by Boussofiane et al. (1991), who state that the number of DMUs should be larger than the product of the number of input and output factors, and Fitzsimmons and Fitzsimmons (1994), who argue that the number of DMUs should be at least twice the sum of the number of input and output factors. The number of DMUs is 15 and the number of input and output factors 3 and 2, respectively, in this study, which satisfies all of the guidelines given above and so, can be deemed appropriate for DEA (Table 2).

**Table 2:** Input and output factors *Source*: From (2012) Samsung Everland

Factors	Classification	Management index
Installation area	Input factor	Fixed cost
Installation cost	Input factor	Fixed cost
Annual repair cost	Input factor	Variable cost
The number of annual users	Output factor	Quantitative index
Customer satisfaction	Output factor	Qualitative index

### Data collection and factor selection

All data for the input and output factors are also extracted from the information provided by the Samsung Everland’s Resort Business Headquarters. In particular, the data for customer satisfaction is, as mentioned above, are based on the results of the 2012 survey conducted by the Resort Business Headquarters. This survey was conducted on 2869 visitors to Everland in 2012. The survey is originally in a 10-point Likert scale but was converted to a 100-point scale for the purposes of this study. The descriptive statistics of the collected data are tabulated in Table 3. One point to note in the data is that, from the actual data, it can be seen that roller coaster-type attractions have broader installation areas, which led to greater installation and repair costs.

**Table 3:** Descriptive statistics of input and output data

Value	Factors				
	Input data			Output data	
	Installation area	Installation cost^a^	Repair cost^a^	The number of users	Customer satisfaction
Max	13,180.00	321.00	0.53	3,090,935.00	92.00
Min	7.00	3.00	0.02	347,233.00	59.00
Average	2182.00	57.80	0.14	1,074,980.53	81.20
Median	282.00	36.00	0.06	939,979.00	84.00
SD	3866.86	81.91	0.17	675,844.02	8.45

^a^Unit: One hundred million and 1py = 3.3058 m^2^

To further verify the appropriateness of the chosen two output factors, a correlation analysis between the two factors, *the number of users* and *customer satisfaction*, was conducted. According to Lewin et al. (1982), output factors in DEA should be selected economically and towards this end, output factors whose correlation is close to 1 can be removed without information loss. Very low correlation was found between the two output factors selected for this study, as listed in Table 4, supporting the validity of the output factors for this study.

**Table 4:** Correlations analysis (output factor)

	The number of users	Customer satisfaction
The number of users		
Pearson correlation	1	0.237
Sig. (2-tailed)		0.394
N	15	15

## Results

**Fig. 3:**
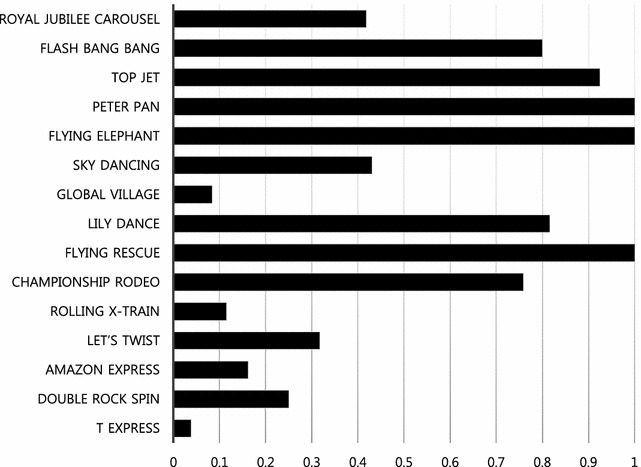
Efficiency score

Using the data collected above, the management efficiency of each attraction, θ value, was calculated under the CCR-DEA model assuming CRS and the results are shown in Fig. 3. Table 5 lists the results for slack variable and reference set analysis. From the results, it was found that the three most efficient DMUs were *Peter Pan*, *Flying Elephant*, and *Flying Rescue* while *T Express* was most inefficient attraction, followed by *Global Village*, *Rolling X*-*Train*, and *Amazon Express*. These four inefficient DMUs recorded efficiencies of 0.2 or lower, which means that more than 80 % of the input factor can be reduced without affecting the output factor. Overall, the analysis shows that rotating-type attractions have higher efficiency and roller coaster-type attractions have relatively lower efficiency. As can be seen in Table 5, the relatively more efficient attractions, *Peter Pan*, *Flying Rescue*, and *Flying Elephant*, served as reference for 12, 8, and 7 times, respectively, and thus, *Peter Pan* is at the top among the benchmarking targets.

**Table 5:** Value of slack variables and reference set

DMU	s_1_^−^	s_2_^−^	s_3_^−^	s_1_^+^	s_2_^+^	Reference
T Express	0	1.5768	0	0	0	7, 11, 12
Double Rock Spin	0	0	0.046	0	10.3324	7, 12
Amazon Express	1572.198	0	0.0033	0	75.8377	12
Let’s Twist	0	5.2471	0	0	0	7, 11, 12
Rolling X-Train	572.4139	0	0.0037	0	0	11, 12
Championship Rodeo	274.6568	29.575	0	0	0	11, 12
Flying Rescue	0	0	0	0	0	7
Lily Dance	0	0	0.0281	0	0	7, 11, 12
Global Village	0	0	0.0201	0	6.4487	7, 12
Sky Dancing	0	1.0615	0	0	2.4781	7, 12
Flying Elephant	0	0	0	0	0	11
Peter Pan	0	0	0	0	0	12
Top Jet	66.111	6.1987	0	0	0	11, 12
Flash Bang Bang	0	0	0.0404	0	0	7, 11, 12
Royal Jubilee Carousel	0	2.3737	0	0	25.8725	7, 12

**Fig. 4:**
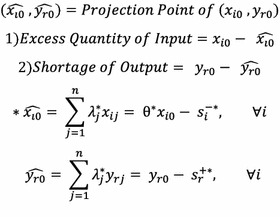
Excess quantity of input and shortage of output

In order to find out the amount of input that needs to be reduced and the amount of output that need to be further produced by the 9 inefficient DMUs in order for them to become reference sets, the excess quantity of input and the shortage of output of the 9 DMUs were calculated using the equation in Fig. 4, following Park (2009). Assuming a constant output, the excess quantity of input and projection point for the DMUs were found to be as listed in Table 7 below. As expected, for efficient attractions such as *Peter Pan*, *Flying Elephant*, and *Flying Rescue*, the projection points are themselves and their excess input quantity is zero. Meanwhile, inefficient attractions like *T Express*, *Amazon Express*, *Rolling X*-*Train*, and *Global Village* or the roller coaster-types have lower numbers of users or lower satisfaction levels compared to amount of input (Table 6).

**Table 6:** Projection point and excess quantity of input

DMU	Input factor					
	Excess quantity of input (%)			Projection point (%)		
	X1	X2	X3	X1	X2	X3
T Express	96.11	96.60	96.05	3.89	3.40	3.95
Double Rock Spin	74.94	74.94	90.91	25.06	25.06	9.09
Amazon Express	95.64	83.72	84.90	4.36	16.28	15.10
Let’s Twist	68.23	82.81	68.33	31.77	17.19	31.67
Rolling X-Train	96.95	88.45	90.10	3.05	11.55	9.90
Championship Rodeo	66.69	84.46	24.00	33.31	15.54	76.00
Flying Rescue	0.00	0.00	0.00	100.00	100.00	100.00
Lily Dance	18.39	18.38	68.42	81.61	81.62	31.58
Global Village	91.53	91.53	95.97	8.47	8.47	4.03
Sky Dancing	56.88	62.19	57.14	43.12	37.81	42.86
Flying Elephant	0.00	0.00	0.00	100.00	100.00	100.00
Peter Pan	0.00	0.00	0.00	100.00	100.00	100.00
Top Jet	41.78	63.87	5.56	58.22	36.13	94.44
Flash Bang Bang	20.04	20.04	73.33	79.96	79.96	26.67
Royal Jubilee Carousel	58.11	64.71	57.63	41.89	35.29	42.37

*X1* installation area, *X2* installation cost, *X3* annual repair cost

That the roller coaster-type attractions are found to have higher input values in terms of installation cost and repair cost implies the possibility of significant correlation among the installation cost, installation size and repair cost. Therefore, a correlation analysis was performed for input factors, the results of which are shown in Table 7. The results confirm that the input factors have significant correlation where the correlation between installation cost and annual repair cost, in particular, is as high as 0.868. While input factors showing high correlation, as in this case, can sometimes be replaced with a different input factor or dropped from analysis altogether, the DEA method used in this study is based on linear programming and does not have the multicollinearity problem that occurs in parametric statistics analysis methods such as regression analysis (Han et al. 2009). Thus, this high correlation between installation cost and annual repair cost does not affect the parameters of this study, but provides interesting insight to the nature of attractions at theme parks.

**Table 7:** Correlations analysis (input factor)

	Installation area	Installation cost	Annual repair cost
Installation area			
Pearson correlation	1	0.658**	0.583*
Sig. (2-tailed)		0.008	0.023
N	15	15	15
Installation cost			
Pearson correlation	0.658**	1	0.868**
Sig. (2-tailed)	0.008		0.000
N	15	15	15
Annual repair cost			
Pearson correlation	0.583*	0.868**	1
Sig. (2-tailed)	0.023	0.000	
N	15	15	15

** Correlation is significant at the 0.01 level (2-tailed)

* Correlation is significant at the 0.05 level (2-tailed)

### Guideline for new theme parks

The DEA on efficiency show that roller coaster type attractions have relatively low efficiency compared to other types of attractions. Then, does this imply that, to achieve better efficiency, future theme parks should not build roller coasters? The answer to this question is, without a doubt, ‘no.’ It is difficult to imagine a theme park without a roller coaster. From the customer’s perspective, while efficient attractions with short waiting time can be important, what is more important are attractions they want to ride again, that is, attractions that deliver high satisfaction. And these attractions are what induce customers to revisit the theme park.

**Fig. 5:**
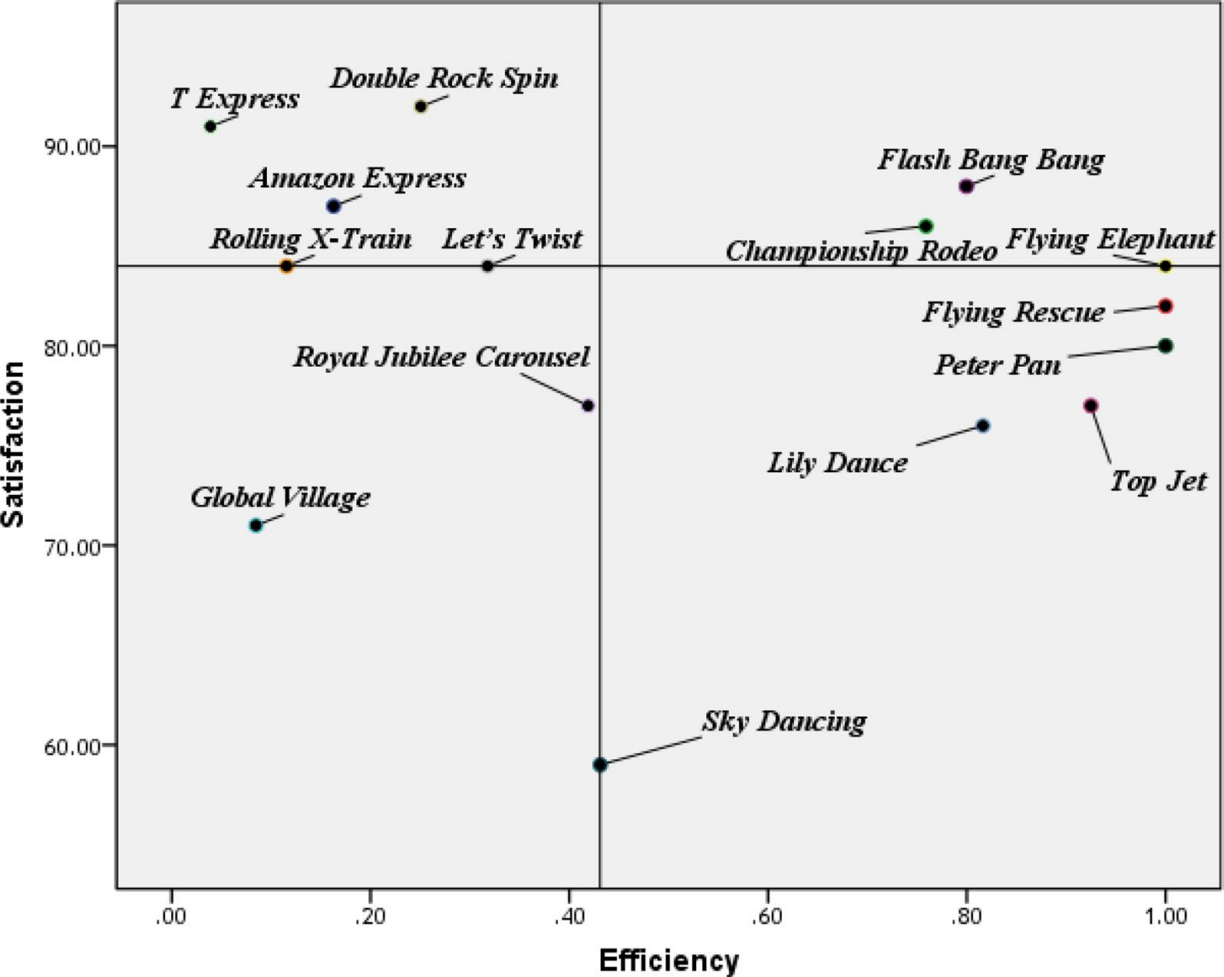
Attraction classification by efficiency and satisfaction

Figure 5 below show how each attraction can be plotted on map of four quadrants that uses score for operation efficiency as the x-axis and that for customer satisfaction as the y-axis, where the scores used for the axes are median values. Roller coaster type attractions such as *Amazon Express*, *Rolling X*-*Train*, and *T Express* fall under quadrant 2, which indicates low operation efficiency but high customer satisfaction. When looking at efficiency and satisfaction together, attractions under quadrant 1 are those which are efficient while delivering high customer satisfaction, and attraction under quadrant 2 are those which are not so efficient but have a brand effect that can bring in visitors. Quadrant 3 shows attractions of low efficiency and low customer satisfaction, and thus, attractions falling under this quadrant should be considered for replacement in existing theme parks and should not be considered for installation in future theme parks. Finally, attractions that fall under quadrant 4 have high operational efficiency but low customer satisfaction, and thus, benchmarking of same-type attractions that deliver high satisfaction is necessary to find ways to increase customer satisfaction.

## Conclusion

This study applies linear programming-based DEA to assess the operational efficiency of individual attractions at theme parks. In consideration of an optimal DMU number, 15 attractions at Samsung Everland were chosen as DMUs. Then, the installation area, installation cost, and annual repair cost were selected as input factors and the number of annual users, and satisfaction as output factors. Actual data for the input and output factors were collected, accounting for fixed cost, variable cost, qualitative factor, and quantitative factor. The results of the DEA showed that the most efficient DMUs were *Peter Pan*, *Flying Elephant*, and *Flying Rescue* whereas the most inefficient attraction was *T Express*. *Global Village*, *Rolling X*-*Train*, and *Amazon Express* also recorded relatively low efficiency levels. Further analysis showed that, to increase efficiency in the inefficient attractions, their installation cost and annual repair cost need to be reduced.

The significance of the present study can be listed as follows. First, the study’s rare use of DEA in analyzing the operational efficiency of theme park attractions and the selection of input and output factors that consider various elements such as fixed cost, variable cost, qualitative factor and quantitative factor serve as a the foundation for utilizing DEA in future studies for the efficient attraction management in theme parks. Second, the projection points and excess quantity of input for each DMU were derived to present attraction-specific efficiency management guidelines, based on which theme parks will be able to manage efficiency-harming factors such as the installation area, installation cost, and annual repair cost.

Third, benchmarking targets were identified for inefficiently-managed attractions. In particular, roller coaster-type attractions were found to be of lower efficiency in general, requiring higher inputs than other types of attractions in terms of installation area, installation cost and repair cost without producing greater outputs. Rather, rotating type-attractions were found more efficient. Lastly, the study analyzes each attraction’s efficiency and satisfaction to discuss what attractions are appropriate for installation in future theme parks. While roller coaster type attractions have relatively low operational efficiency, they are able to deliver high customer satisfaction and thus carry a brand effect that increases theme park customers’ revisit intention.

One of the limitations of this study is that its investigation is specific to attractions at Samsung Everland, a South Korean theme park. Subsequent studies should look into more diversified DMUs (for instance, theme parks in other countries) and consider more varied input factors accordingly. Another suggestion for future research is to set a weighted value for each variable. In the case of Samsung Everland, while the present study found *T Express* the most inefficient attraction of all, its customer satisfaction ranked 2nd among the 15 DMUs. Thus, follow-up studies will benefit from considering the use of additional analysis methods such as analytic hierarchy process (AHP) to investigate the weighted value for each input and output factor. Finally, it is difficult to make effective marketing strategies without understanding the reason behind customers’ visits (Fodness 1994), however, due to limitations in the methodology used, this study was unable to reflect this aspect within its parameters. It is hoped that future studies will consider various factors that may provide the reasons for customers to visit the theme park, and enrich the research on theme park management.
